# Construction of miRNA-miRNA networks revealing the complexity of miRNA-mediated mechanisms in trastuzumab treated breast cancer cell lines

**DOI:** 10.1371/journal.pone.0185558

**Published:** 2017-10-05

**Authors:** Emine Ezel Cilek, Hakime Ozturk, Bala Gur Dedeoglu

**Affiliations:** 1 Biotechnology Institute, Ankara University, Ankara, Turkey; 2 Department of Computer Engineering, Bogazici University, Istanbul, Turkey; University of South Alabama Mitchell Cancer Institute, UNITED STATES

## Abstract

Trastuzumab is a monoclonal antibody frequently used to prevent the progression of HER2+ breast cancers, which constitute approximately 20% of invasive breast cancers. microRNAs (miRNAs) are small, non-coding RNA molecules that are known to be involved in gene regulation. With their emerging roles in cancer, they are recently promoted as potential candidates to mediate therapeutic actions by targeting genes associated with drug response. In this study we explored miRNA-mediated regulation of trastuzumab mechanisms by identifying the important miRNAs responsible for the drug response via homogenous network analysis. Our network model enabled us to simplify the complexity of miRNA interactions by connecting them through their common pathways. We outlined the functionally relevant miRNAs by constructing pathway-based miRNA-miRNA networks in SKBR3 and BT474 cells, respectively. Identification of the most targeted genes revealed that trastuzumab responsive miRNAs favourably regulate the repression of targets with longer 3’UTR than average considered to be key elements, while the miRNA-miRNA networks highlighted central miRNAs such as hsa-miR-3976 and hsa-miR-3671 that showed strong interactions with the remaining members of the network. Furthermore, the clusters of the miRNA-miRNA networks showed that trastuzumab response was mostly established through cancer related and metabolic pathways. hsa-miR-216b was found to be the part of the most powerful interactions of metabolic pathways, which was defined in the largest clusters in both cell lines. The network based representation of miRNA-miRNA interactions through their shared pathways provided a better understanding of miRNA-mediated drug response and could be suggested for further characterization of miRNA functions.

## Introduction

With at least 1.3 million new cases per year, breast cancer is the most frequently seen cancer type among women worldwide. Despite the decreasing mortality rate in our decade, it is still a life threatening disease with different histological and molecular subtypes [1]. The majority of poor clinical outcome is usually related to the development of metastasis with drug resistance, which is mostly seen in HER2+ metastatic breast cancers [2,3]. So far, the humanized anti-HER2 monoclonal antibody, trastuzumab (Herceptin), has been a key component used for the treatment of HER2+ early stage cancers. However, the response rate to trastuzumab monotherapy is only around 35% and the development of resistance to the agent after the first year of treatment is still an emerging problem[2,4]. As a result, identification of the mechanisms underlying the trastuzumab antitumour activity still keeps its importance for the discovery of new combinational and single agent therapies as well as the novel treatment strategies [4–6].

microRNAs (miRNAs) are endogenous small non-coding RNAs approximately 22 nucleotides in length that play regulatory roles in gene expression by mediating mRNA cleavage or translational repression [7]. A single miRNA can target several genes, more than a hundred mRNAs in average. 60% of whole human protein coding genes are predicted to have miRNA-binding sites in their 3’ untranslated regions (3’UTRs). Together with the number of identified miRNAs running into thousands, they form one of the most abundant classes of the gene-regulatory systems in the cell [8]. Thus, any deregulation of the miRNAs might cause a major disruption in the gene regulation mechanisms of the cell that might even lead to the cancerous phenotypes [9]. It has been shown that miRNAs are deregulated in breast cancer and various types of other human cancers [10,11]. Since miRNAs might have effective roles in the progress of diseases, they are likely to become potential therapeutic targets for cancer as well. A therapeutic benefit could be provided by modulating the expression levels of miRNAs in the disease state [12]. A recent study has showed that level of miR-210 in plasma might be associated with trastuzumab resistance in patients [13]. It was followed by other findings indicating the effect of trastuzumab on the expression of miRNAs, however, these studies only have focused on the oncogenic and tumor suppresor functions of the individual miRNAs in trastuzumab sensitive or resistance cell lines [14–19]. Unfortunately they fail to explain the complexity of miRNA mediated drug mechanisms due to the absence of information on the regulatory interaction networks.

Network analysis is one of the widely adopted approaches to discover driver genes and pathways in biological systems [20]. Recent studies have shown that miRNA networks have synergistic roles in the regulation of pathological conditions. If two miRNAs interact with each other in a network, they are more likely to regulate the pathways and target genes with similar functions in the same disease [21–23]. Therefore, investigation of the interactions between miRNAs on the network models might provide significant insights to explain the complex regulatory mechanisms in the drug treatment [24].

In this study, our aim was to uncover the underlying mechanisms of trastuzumab treatment by elucidating the miRNA-regulatory networks in breast cancer cell lines. For this purpose, we first performed a microarray miRNA profiling in trastuzumab treated cells (Geo Accession Number: GSE104076)to find out responsive miRNAs. We constucted a miRNA-miRNA network model that was capable of the visualization of functionally relevant miRNA pairs, where miRNAs were represented as nodes and the edges represented the targeted pathways or biological processes. The application of network analysis to trastuzumab responsive miRNAs revealed the genes that were highly favored by the miRNAs, such as KSR2 (kinase suppressor of ras 2); MDM4 (p53 regulator); UBE2W ubiquitin conjugating enzyme E2 W); CADM2 (cell adhesion molecule 2); ARL15 (ADP ribosylation factor like GTPase 15). These genes were known to involve in the regulation of MAPK signaling pahtway, cell cycle, metabolism of proteins, cell-cell communication of cancer cells. Functionally relevant miRNA pairs were found in the networks based on their shared pathways and biological processes, which helped us to find out the prominent miRNA-mediated mechanisms in trastuzumab treatment. We suggest that uncovering the synergistic effects between miRNAs might deepen our knowledge about their potential roles and their interacting molecular targets in the drug treatment and provide new insights into the trastuzumab treatment in system-wide level.

## Materials and methods

### Cell lines and reagents

HER2+ breast cancer cell lines BT474 (HTB-20) representing Luminal B (ER+; PR+, HER2+; ductal carcinoma) subtype and SKBR3 (HTB-30) representing HER2+ subtype (ER-; PR-; HER2+; adenocarcinoma) of breast cancer were purchased from the American Type Culture Collection (ATCC) [25,26], which were known to well characterized in previous studies and decribed highly sensitive to trastuzumab treatment [27,28]. SKBR3 cells were maintained in Mc Coy’s 5A medium (Lonza) with L-glutamine containing 10% fetal bovine serum (FBS), 1% penicillin-streptomycin. BT474 cells were maintained in RPMI 1640 medium with L-glutamine (Lonza) supplemented with 10% FBS, 1% penicillin-streptomycin and 2% bovine insulin. The cell lines were cultured in a humidified air supplemented with 5% CO_2_ at 37°C. Trastuzumab was (kindly gifted by Prof. Dr. Hakan Gürdal from the Department of Pharmacology in Medical School of Ankara University) dissolved in phosphate-buffered saline (PBS) (stock concentration of 300 mg/mL) and stored at 4°C.

### WST-1 assay and trastuzumab treatment

The WST-1 assays (Roche Applied Science) were performed to see the sensitivity of the cells to trastuzumab. The assays were maintained according to the manufacturer's instructions. BT474 and SKBR3 cells were plated as 5x10^3^ cells per well in 96-well plates. After 24 hours, the cells were exposed to trastuzumab at different concentrations as 0.05, 0.1, 0.5, 2, 6, 30, 60, μg/ml and they were kept in drug treatment for 6 days. The culture media containing trastuzumab were replaced every 72 hours. On day 6, 10 μl of WST-1 reagent was added into each well. After 2 hours of incubation at 37°C, the absorbance at 450 nm was measured by a spectrophotometric reader (Perkin Elmer’s Victor Plate Reader). The half maximal inhibitory concentration (IC_50_) was calculated by using Graphpad Prism 6 Program.

For further experiments, SKBR3 and BT474 cells were plated at a starting density of 2 x 10^6^ in 100 mm cell culture plates. BT474 and SKBR3 cells were treated with 6 μg/mL trastuzumab and/or PBS as control within two biological replicates for 144 hours. The media were changed in every 72 hours.

### RNA isolation and miRNA profiling by microarray analysis

Total RNA was isolated with the TRIzol reagent (Invitrogen) according to the manufacturer's instructions. Absorption at 260 nm and 280 nm was measured for the determination of RNA purity. The integrity of the RNAs was determined on the gel electrophoresis by checking out the 18S/28S ribosomal RNA ratios. Hybridization was performed in Human miRNA Microarray, Release 19.0, 8x60K (G4870A, Agilent Technologies) platform, which was an array designed from miRBase version 19 containing 2006 miRNAs. Each miRNA sequence was represented by probes replicated at least 30 times on the array. Spike-in control solutions were first prepared and total RNA (100 ng) from each sample was dephosphorylated with calf intestine alkaline phosphatase at 37°C for 30 min. It was followed by a denaturation step that includes the incubation of samples in DMSO at 100°C for 10 minutes. Samples were then labeled with Cyanine3-pCp by using T4 RNA Ligase at 16°C for 2 hours. They were mixed with 10x blocking agent and 2x Hi-RPM hybridization buffer (Agilent Technologies), and hybridizations were performed at 55°C with rotation at 20 rpm. After the washing step, the arrays were scanned in Roche Nimblegen instrument by using Agilent Scan Control software. The data were acquired using Agilent Feature Extraction software for miRNA microarray, generating a GeneView file that contained summarized signal intensities for each miRNA by subtracting the background after combining the intensities of replicate probes.

### Microarray data analysis

Two biological replicates were used for each treated and untreated cells. A total of 4 samples from BT474 cells (2 from trastuzumab treated and 2 from PBS treated) and 4 samples from SKBR3 cells (2 from trastuzumab treated and 2 from PBS treated) were analyzed. BRB Array Tools 4.3.2. stable release was used for the normalization and statistical analysis. Bioconductor packages were used for the normalization and statistical comparisons. The data were normalized by Quantile normalization method [29]. The stastistical comparisons were done by using t-test based class comparison function of BRB Array Tools, which is called “between group of arrays (BGA)”. In order to find out the differential expressed miRNAs between the treated and non-treated cell lines, p-value less than 0.05 and fold changes more than 2 were used as cut-off values.

### Target prediction of trastuzumab responsive miRNAs

Two different target prediction algorithms (DIANA-microT-CDS v5.0 and TargetScan v71) were used for *in silico* target identification of the differentially upregulated and downregulated miRNAs in both cell lines [30,31]. While DIANA-microT-CDS uses thermodynamic-based algorithm, TargetScan relies on a seed complementarity model for the target prediction [32]. Target genes of each responsive miRNA were investigated in both databases. Top 200 of the predicted targets were selected in TargetScan V7 and TargetScan V7.1, a cut-off value of 0.7 was applied to DIANA-microT-CDS database for the prediction of target genes. The intersection of the predicted targets from two algorithms were used to increase the sensivity of the target prediction [33]. The overlapping target genes were listed for further analysis.

### Venny analysis

A Venn Diagram analysis; “Venny http://bioinfogp.cnb.csic.es/tools/venny/index.html” was performed to determine the overlapping target gene sets, which were defined as common predicted targets in both DIANA-microT-CDS and Targetscan tools. The overlapping lists were determined as the final target gene lists for each responsive miRNA.

### Pathway enrichment analysis of the target genes

To better understand the functional characters of miRNA-miRNA pairs, a KEGG pathway enrichment analysis was performed for the final target gene lists by using WebGestalt tool [34,35]. The overlapping target gene list of each responsive candidate miRNA were uploaded into the WebGestalt tool. As the default setting the minimum number of genes was adjusted to 2 from the list that was required for a pathway to be considered. The adjusted p-value of each enriched pathway was calculated with the method of Benjamini and Hockberg and the statistically enriched pathways were obtained using hypergeometric test (p-value < 0.05). To construct the networks, KEGG pathway terms were used and they were collected in a list (e.g. Metabolic pathways—hsa01100).

### Network construction

The networks presented in this study were built by using trastuzumab responsive miRNA profiling data acquired from our study (Geo Accession Number: GSE104076). For the construction of the networks, we classified the trastuzumab responsive miRNAs in two groups as “responsive miRNAs in SKBR3” and “responsive miRNAs in BT474”. To find out the functional features of miRNA pairs, a pathway enrichment analysis was performed using the final target gene lists and they were used to connect the miRNA pairs in the network.

For each miRNA pair, the corresponding significance value was calculated using hypergeometric distribution [36].

$$P=1-\overset{x-1}{\underset{i=0}{\sum }}\frac{\left({}_{i}^{m}\right)\left({}_{n-i}^{N-m}\right)}{\left({}_{n}^{N}\right)}$$

where N was the total number of genes/pathways, n was the number of genes/pathways that were regulated by one responsive miRNA, m was the number of genes/pathways that were regulated by the other miRNA, and x was equal to the number of common genes/pathways for both miRNAs. The miRNA pairs were considered as significant and included into the network if their p-value was p<0.05. In the pathway-based analysis N was set to the number of KEGG pathways (400 pathways) and in the gene-based analysis N was set to the number genes in the WebGestalt NCBI Gene data source (28.000 genes).

The networks were created in the form of interaction lists in which each line represented an interaction between two miRNAs, including the type and the weight of the interaction. Visual Studio 2010 was used to implement the proposed network models. Cytoscape Version 3.2.0 [37] was used to visualize and analyze the networks. To identify central nodes with high degrees, Network analyzer plugin of Cytoscape was utilized. The most central node of the network indicated the node with the most interaction in terms of degree and the size of the nodes represented their degree values (degree centralities). The upregulated miRNAs were visualized as red nodes whereas the downregulated miRNAs were visualized as green nodes.

The networks presented in this study were designed as homogenous networks to define the relationships of functionally relevant miRNAs. The miRNA-miRNA network model was inspired from a recent study where the proteins were connected to each other through their shared ligands to construct a protein-protein network [38]. Similarly, we built a miRNA-miRNA interaction network by linking miRNAs through their shared enriched pathways or biological processes.

**Fig 1:**
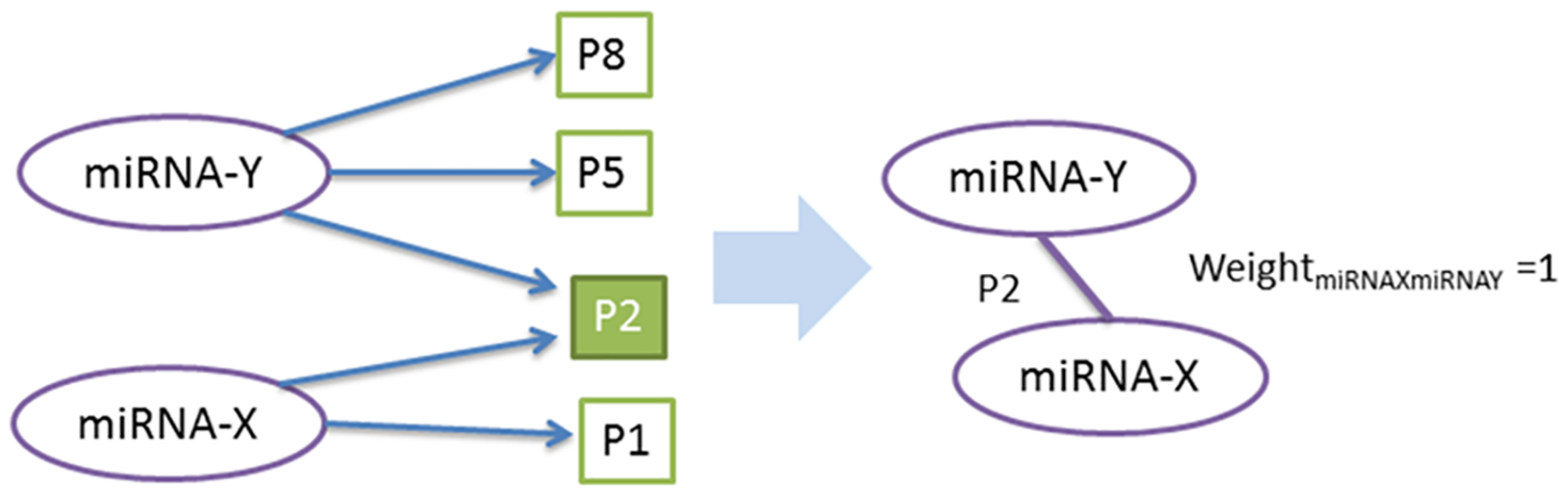
An example of the network model in the study. miRNA X was known to target the pathways P1 and P2; miRNA Y, on the other hand, was related to the pathways P2, P5 and P8. The pathway based network model helped us to connect miRNA X and miRNA Y through their common pathway P2. The weight of the edge between miRNA X and miRNA Y was set to be one, since they shared one single pathway. The corresponding significance value was calculated using hypergeometric distribution for each miRNA pair. The less significant miRNA pairs with p-value larger than 0.05 were filtered out of the network.

In our model, the nodes of the network represented miRNAs and the edges were either the shared biological processes or pathways between a pair of miRNAs. Each edge in the network had a weight indicating the number of the shared features. For instance; two virtual miRNAs; miRNA X and miRNA Y were used to figure out their functional roles. miRNA X was known to relate with the pathways P1 and P2; miRNA Y, on the other hand, was related to the pathways P2, P5 and P8. The pathway based network model allowed us to connect miRNA X and miRNA Y through their common pathway P2. The weight of the edge between miRNA X and miRNA Y was equal to one, since they only shared a single pathway (Fig 1). Through the network analysis the most targeted genes were also determined based on the total number of miRNAs regulating them in the network.

#### Clustering of the trastuzumab responsive miRNA-miRNA pairs in the pathway based network models

Cluster analysis of the network reveals cliques of nodes that we expect to have similar properties since they are tightly connected. In this study, we performed the cluster analysis using “Clustermaker2” plugin of Cytoscape [39]. Since the networks were implemented as undirected weighted networks, the sub-miRNAs or cliques were found out by “Markov Cluster (MCL) Algorithm” to figure out the contribution of edges and the power of links between their interacted nodes [40]. An automatic edge threshold was applied to the network to get more specific cluster families.

### Functional validation of the most targeted genes

A functional validation was performed with the top targeted gene sets in the networks to understand whether they were related to breast cancer or not. The validation was done using The Cancer Genome Atlas (TCGA) Breast Cancer gene expression dataset (AgilentG4502A_07_3 array), which was provided to access publicly as a part of the UCSC Cancer Genome Browser [41]. This dataset uses the log2 lowess normalized ratio of sample signal to reference signal (cy5/cy3) collapsed for each gene. The top 30 targeted genes of responsive miRNAs in BT474 and SKBR3 cells were searched for their expression values in TCGA Breast Cancer data sets by using “UCSC Xena http://xena.ucsc.edu” program.

### Statistical analysis

We performed the statistical analyses with Graphpad Prism 6, Microsoft Excel 2010, Visual Basic 2010 and BRB Array Tools 4.3.2. programs. The half maximal inhibitory concentration (IC_50_) was calculated by using dose-response curves in Graphpad Prism 6 Program. A two sample t-test was used to determine the differentially expressed miRNAs in microarray profiling by using BRB Array Tools 4.3.2. release. The significances of the miRNA networks were found out by using a hypergeometric distribution and *P*-value<0.05 was defined as the cut-off value for the significant miRNA interactions.

## Results

### Trastuzumab responsive miRNAs in SKBR3 and BT474 cell lines

131 upregulated and 134 downregulated miRNAs were identified in BT474 cells, while 104 upregulated and 98 downregulated miRNAs were found to be differentially expressed in SKBR3 cells (Table 1) (S1 Table and S1 and S2 Figs). The differentially expressed miRNAs were described as “responsive miRNAs” in the trastuzumab treatment (S1 Table). miRNAs that did not have the overlapped predicted target lists obtained by both DIANA-microT-CDS and TargetScan tools or miRNAs had the intersected targets but did not show functional enrichment in any pathway or biological process were omitted from the data source. 57 miRNAs were omitted from BT474 dataset, while 49 miRNAs were omitted from SKBR3 dataset (S2 Table).

**Table 1:** 20 responsive miRNAs with the greatest difference in expression levels in trastuzumab treated SKBR3 and BT474 cell lines.

MicroRNA ID	Gmean BT474 Trastuzumab	Gmean BT474 PBS	Fold-change	p-value*
hsa-miR-34c-3p	76.9	10	7.69	0.000495
hsa-miR-588	85.79	14.4	5.96	0.001264
hsa-miR-580	58.94	10	5.89	0.001384
hsa-miR-374a-3p	175.49	29.84	5.88	0.001392
hsa-miR-616-3p	62.19	10.83	5.74	0.001721
hsa-miR-3124-3p	166.41	29.76	5.59	0.002031
hsa-miR-5088	307.06	56.29	5.46	0.002344
hsa-miR-541-5p	97.02	19.1	5.08	0.00259
hsa-miR-493-3p	83.25	16.82	4.95	0.002595
hsa-miR-551b-5p	83.11	17.34	4.79	0.002642
hsa-miR-466	33.72	299.95	-8.90	0.048349
hsa-miR-5692a	12.15	102.61	-8.45	0.049597
hsa-miR-99a-3p	18.39	123.48	-6.71	0.049275
hsa-miR-379-3p	29.91	200.03	-6.69	0.049586
hsa-miR-3671	11.07	62.79	-5.67	0.048871
hsa-miR-937-3p	13.98	77.8	-5.57	0.048947
hsa-miR-876-5p	16.9	89.68	-5.31	0.048314
hsa-miR-1228-5p	17.77	90.27	-5.08	0.048149
hsa-miR-300	20.41	102.78	-5.04	0.04733
hsa-miR-6502-3p	19.4	97.29	-5.01	0.047564
MicroRNA ID	Gmean SKBR3 Trastuzumab	Gmean SKBR3 PBS	Fold-change	p-value*
hsa-miR-580	185.53	11.47	16.18	0.003577
hsa-miR-338-5p	125.93	11.94	10.54	0.005305
hsa-miR-5196-5p	215.5	24.32	8.86	0.00041
hsa-miR-4768-3p	129.57	16.88	7.68	0.001565
hsa-miR-92b-5p	78.06	10.56	7.39	0.004378
hsa-miR-4754	199.53	27.21	7.33	0.001553
hsa-miR-890	67.76	10.02	6.76	0.004637
hsa-miR-6716-5p	97.1	15.48	6.27	0.008135
hsa-miR-613	149.1	23.91	6.24	0.035063
hsa-miR-5090	527.56	85.64	6.16	0.001562
hsa-miR-200a-3p	24.74	520.28	-21.03	0.041457
hsa-miR-339-3p	43.85	763.4	-17.41	0.004607
hsa-miR-345-5p	18.87	296.55	-15.72	0.001076
hsa-miR-19a-5p	24.05	233.07	-9.69	0.030978
hsa-miR-4760-5p	11.64	108.72	-9.34	0.021372
hsa-miR-3684	20.84	162.31	-7.79	0.002313
hsa-miR-190a	34.15	254.61	-7.46	0.043939
hsa-miR-514a-5p	20.39	139.41	-6.84	0.001464
hsa-miR-3976	25.61	161.05	-6.29	0.028377
hsa-miR-10b-5p	39.53	239.88	-6.07	0.008059

Gmean BT474 trastuzumab and BT474 PBS, Geometric mean values of signal intensities in trastuzumab and PBS treated BT474 cell lines; Gmean SKBR3 trastuzumab and SKBR3 PBS, Geometric mean values of signal intensities in trastuzumab and PBS treated SKBR3 cell lines

*p-values calculated using a two sample ttest (random variance model).

By omitting aforementioned miRNAs we obtained a final list of 208 miRNAs in BT474 cells and 153 miRNAs in SKBR3 cells for the construction of the networks (S3 Table).

### Network analysis

First, less significant miRNA pairs with p-values larger than 0.05 were filtered based on the hypergeometric distribution. Then the final datasets with more significant miRNA pairs were used for the network construction. The networks were constructed for BT474 cell line and SKBR3 cell line, respectively. Each network comprised the upregulated and downregulated responsive miRNAs together. Table 2 illustrates the detailed information about the final data sets including the number of statistically significant miRNAs as well as the number of total unique genes and pathways that those miRNAs were related to.

**Table 2:** Detailed information of the pathway based miRNA-miRNA network dataset in the trastuzumab treated BT474 and SKBR3 cell lines.

Data	Total number of the interacting miRNAs in the network	Total number of the enriched/shared pathways*	Total number of the shared target genes*
BT474	150	152	4140
SKBR3	73	146	2433

*Shared genes/pathways by at least two miRNAs

Degree centrality helped us to identify the most important nodes in the network in terms of number of interactions, which were defined based on the shared pathways or biological processes that they were involved in while the most targeted genes by miRNAs were identified based on their frequencies in the networks. Cluster analyses enabled us to focus on the functionally relevant miRNA pairs and their interactions.

#### Identification of the most targeted genes may reveal potential key players in miRNA-regulatory mechanisms

4140 genes were found to be targeted by at least two miRNAs in BT474 cells, while 2433 genes were targeted in SKBR3 cells. The most targeted gene in trastuzumab treated BT474 cells was FAM9C, which was predicted to be targeted by 19 different miRNAs. It was followed by SAMD12 and UBE2W which were potentially regulated by 16 miRNAs and 15 miRNAs, respectively. Among the top 30 targeted genes, MDM4 (p53 regulator), CAMD2 (cell adhesion molecule), KSR2 (kinase suppressor 2 of RAS) and EREG (epiregulin) were the most prominent targets that have important roles in the tumor development.

In trastuzumab treated SKBR3 cells, SAMD12 was also one of the most targeted genes, which was potentially regulated by 12 miRNAs. ARL15 and TFRC were the following ones with 10 miRNAs. PFN2 (profilin 2), BTG1 (BTG anti-proliferation factor 1), LPP (LIM domain containing preferred translocation partner in lipoma) and CDK6 (cyclin dependent kinase 6) came to prominence as significant genes that have important effects in the cancer progression (Table 3).

**Table 3:** Top 30 genes targeted by responsive miRNAs in trastuzumab treated BT474 and SKBR3 cells that were used for the functional validation.

Gene ID	Gene Frequency in BT474*	Gene ID	Gene Frequency in SKBR3*
FAM9C	19	SAMD12	12
SAMD12	16	TFRC	10
UBE2W	15	ARL15	10
CCDC38	13	CLLU1	9
MDM4	13	CADM2	9
CADM2	13	LPP	9
KSR2	13	PFN2	8
CLLU1	13	STC1	8
INO80D	13	TMEM154	8
TFRC	12	KLF12	8
FZD3	12	BTG1	8
OSTN	12	C22ORF46	8
CYP3A5	12	RAB27B	8
LPP	12	GNG12	8
FAM222B	11	IMPG1	8
TMEM212	11	REEP3	7
GABRA4	11	MLANA	7
PI15	11	PI15	7
BTLA	11	PRPF38A	7
ZFP36L1	11	DST	7
GYPA	11	C9ORF170	7
RAB30	11	FAM9C	7
CHTF8	10	YWHAG	7
EREG	10	CDK6	7
ZNF286B	10	FAM169A	7
CYB561D1	10	CPLX2	7
ZNF676	10	RAB3B	7
ZNF680	10	S100A7A	7
C5ORF28	10	C16ORF87	7
GNG12	9	TMEM170B	7

* Gene frequency; total number of the potential miRNAs that might target the particular gene seen in the networks

Recent studies indicate that targets with longer 3’UTR than average are tend to be more evolutionarily conserved and they might be key hub genes in the regulation of miRNAs [24,42]. Hence, we investigated our most targeted genes for the length of their target sites by using TargetScan to confirm their potential of being prominent targets in the regulation of trastuzumab responsive miRNAs. Indeed, majority of the genes with the highest frequency in the networks were found to have longer miRNA target sites than average (Table 4).

**Table 4:** Lengths of miRNA target sites for the most targeted genes in the trastuzumab miRNA-miRNA networks.

Gene ID	Gene Frequency in BT474*	Target site length (3’UTR length)**
SAMD12	19	8254 nucleotides
FAM9C	16	5260 nucleotides
UBE2W	15	7882 nucleotides
CCDC38	13	2341 nucleotides
MDM4	13	9420 nucleotides
CADM2	13	7664 nucleotides
Gene ID	Gene Frequency in SKBR3*	Target site length (3’UTR length)**
SAMD12	12	8254 nucleotides
ARL15	10	2650 nucleotides
TFRC	10	4699 nucleotides
CLLU1	9	2809 nucleotides
CADM2	9	7664 nucleotides
LPP	9	16193 nucleotides

*3’UTR; 3’untranslated region of transcripts

** Gene frequency; total number of the potential miRNAs that might target the particular gene seen in the networks

**Fig 2:**
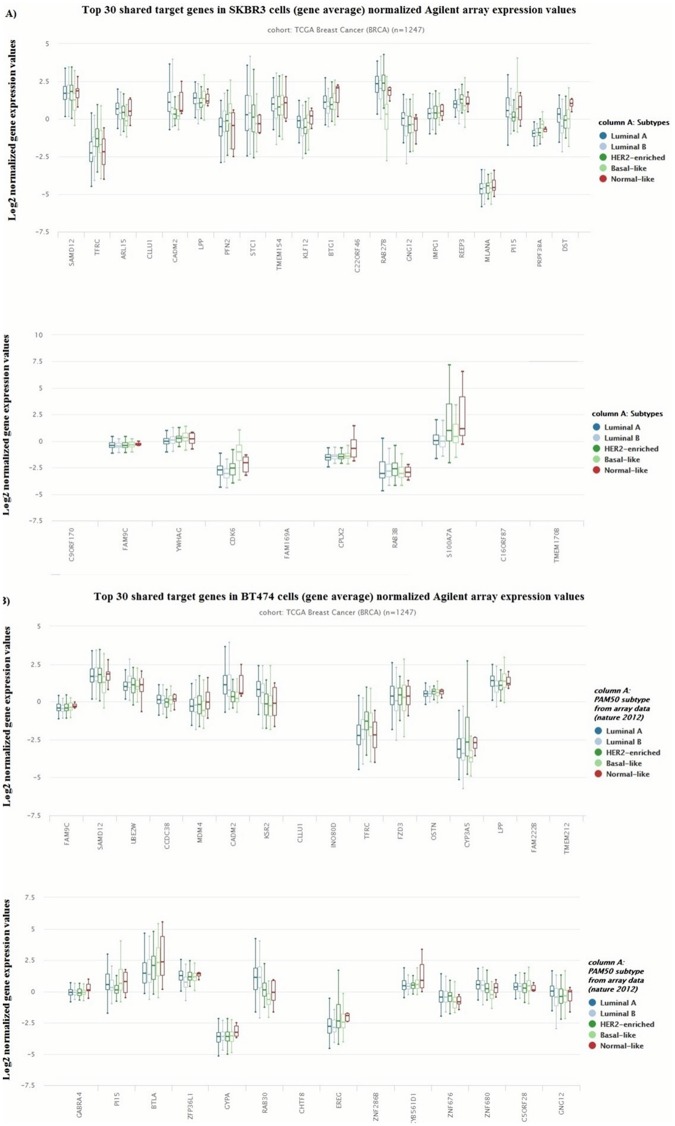
The expression values of top 30 targeted genes in the network in TCGA breast cancer data. (A-B) TCGA breast invasive carcinoma (BRCA) gene expression (AgilentG4502A_07_3 array) was obtained by using 1247 samples in total. The expression values of the genes were given according to molecular subtypes of breast cancer (Luminal A, Luminal B, HER2+, Basal Like and normal like). The expression levels were indicated as in log2 lowess normalized ratio of sample signal to reference signal (cy5/cy3) collapsed for each gene. In order to view the differential expression between samples more easily, the default view was set to center each gene or exon to zero by independently subtracting the mean of each gene or exon on the fly. The data sets were visualized by using Xena Browser.

In addition, we searched for the expression values of each target gene in breast cancer by using The Cancer Genome Atlas (TCGA) Breast Cancer gene expression dataset (AgilentG4502A_07_3 array) [41]. It included 1247 samples in total. Most of the target genes were defined to be differentially expressed in breast cancer. 6 out of 30 most frequent genes in SKBR3 cells were found to be significantly upregulated or downregulated in different breast cancer subtypes (Fig 2A), while 8 out of 30 most frequent genes in BT474 were defined to be differentially expressed in TCGA breast cancer data (Fig 2B).

#### The pathway based miRNA-miRNA networks in SKBR3 and BT474 cells indicate functionally related miRNA pairs

**Fig 3:**
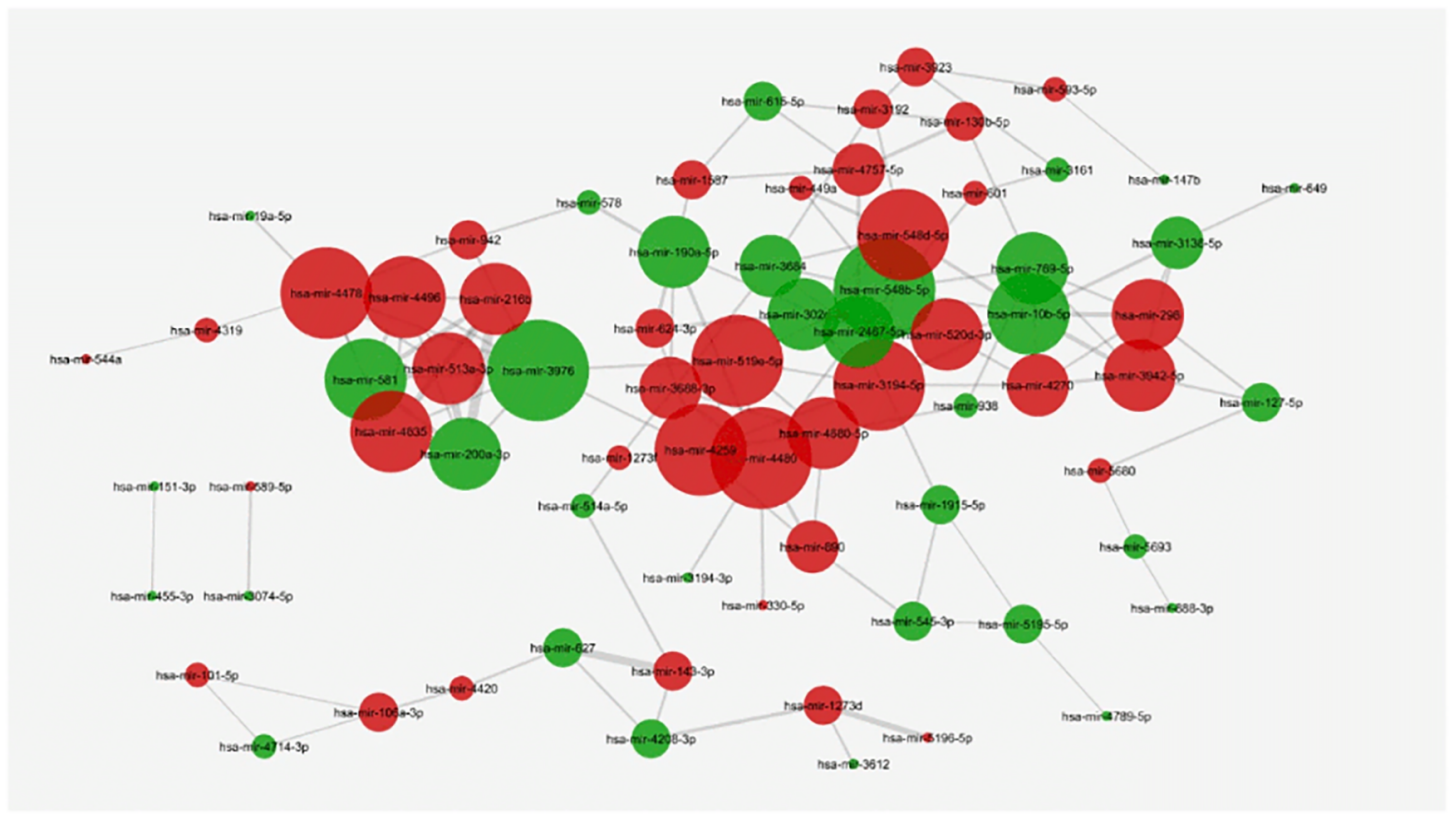
The miRNA-miRNA network in SKBR3 cell. In SKBR3 cells, 73 trastuzumab responsive miRNAs are found to be functionally relevant with each other. Each node represents a responsive miRNA and the nodes are sized by their degree centrality scores. hsa-miR-3976, hsa-miR-548b-5p and hsa-miR-3194-5p are identified to be the most central nodes with the degree scores of 9 and 8 (P<0.05 for each miRNA pair, red nodes:upregulated miRNAs, green nodes: downregulated miRNAs).

In trastuzumab treated SKBR3 cells, 146 pathways were targeted by at least two miRNAs. These pathways connected 73 miRNAs with each other. hsa-miR-3976 was one of the most central nodes together with hsa-miR-548b-5p and hsa-miR-3194-5p in the network with the degree centrality scores of 9 and 8 (Table 5) (S4 Table). The downregulated and upregulated miRNAs equally contributed to the most central nodes (Fig 3). We also identified hsa-miR-3976, hsa-miR-190a and hsa-miR-10b-5p among the top responsive miRNAs with the greatest difference in expression levels according to microarray profiling in trastuzumab treated SKBR3 cells (Table 1).

**Table 5:** 15 central nodes with the highest degree scores in trastuzumab treated SKBR3 and BT474 miRNA networks. All the nodes values were statistically significant with P-value <0.05.

Node name (miRNA ID)	Degree centrality score in SKBR3 network	Node name (miRNA ID)	Degree centrality score in BT474 network
hsa-miR-3976	9	hsa-miR-3671	19
hsa-miR-548b-5p	9	hsa-miR-4474-5p	16
hsa-miR-4480	9	hsa-miR-559	15
hsa-miR-548d-5p	8	hsa-miR-4517	15
hsa-miR-3194-5p	8	hsa-miR-485-5p	14
hsa-miR-4259	8	hsa-miR-558	13
hsa-miR-519e-5p	8	hsa-miR-29b-2-5p	13
hsa-miR-4478	8	hsa-miR-150-3p	12
hsa-miR-4496	7	hsa-miR-411-3p	12
hsa-miR-4635	7	hsa-miR-526b-3p	12
hsa-miR-581	7	hsa-miR-93-3p	11
hsa-miR-10b-5p	7	hsa-miR-551b-5p	10
hsa-miR-769-5p	6	hsa-miR-5579-3p	10
hsa-miR-190a-5p	6	hsa-miR-146b-5p	10
hsa-miR-216b	6	hsa-miR-3121-3p	10

**Fig 4:**
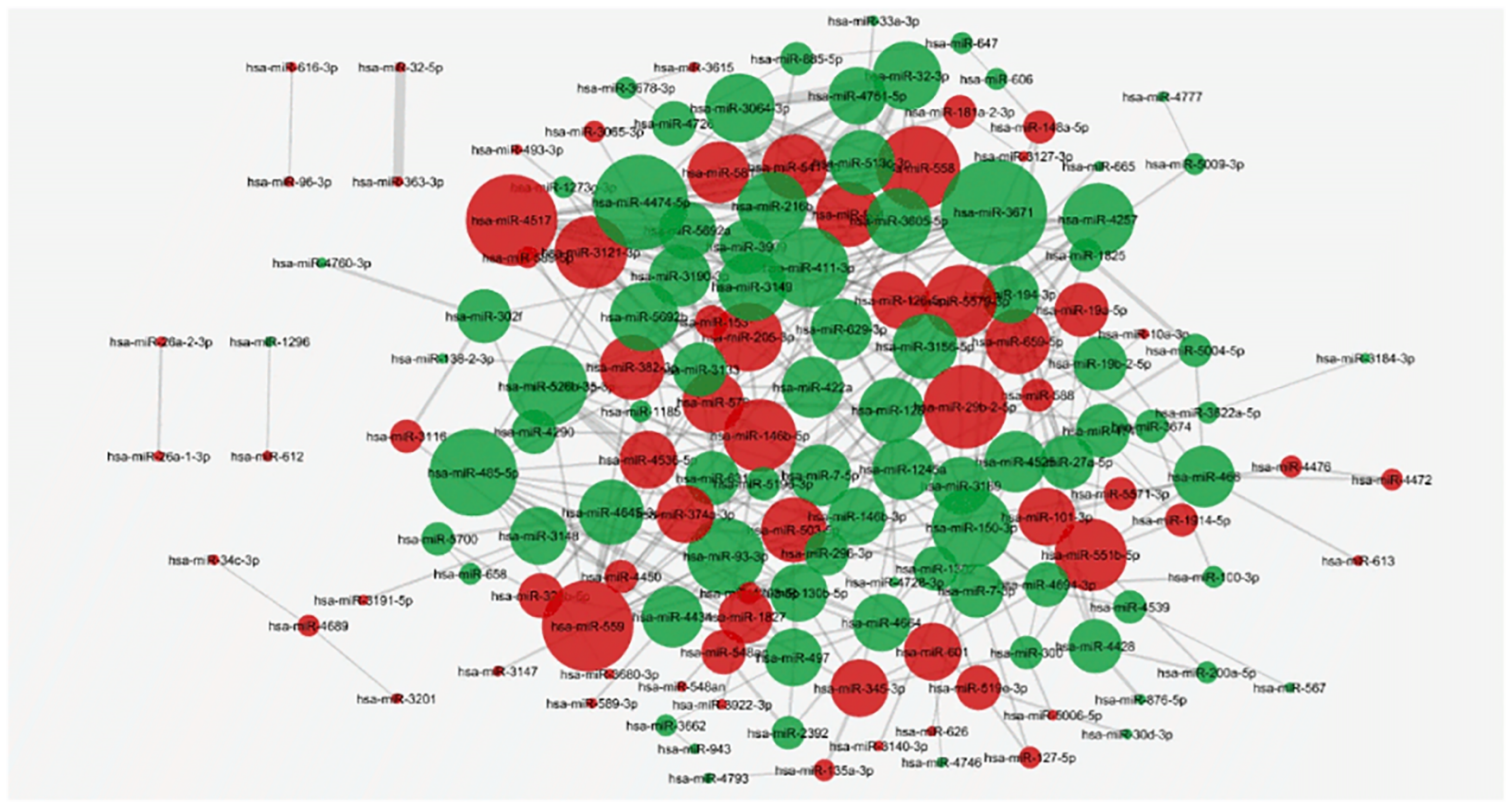
The miRNA-miRNA network in BT474 cell. In BT474 cells, 150 trastuzumab responsive miRNAs are defined as functionally relevant with each other. Each node represents a responsive miRNA and the nodes are sized by their degree centrality scores. hsa-miR-3671 is the most central node in the network with the degree centrality score of 19 (P<0.05 for each miRNA pair, red nodes:upregulated miRNAs, green nodes: downregulated miRNAs).

In trastuzumab treated BT474 cells, 152 pathways were targeted by at least two miRNAs. 150 miRNAs were significantly interacted in the network. Majority of the nodes were defined to have higher degree centralities compared to SBKR3 network, which showed that the responsive miRNAs involved with certain biological processes in larger numbers and they were tightly interacted with each other in BT474 cells (Fig 4). hsa-miR-3671 was the most central node with the degree score of 19. It was followed by hsa-miR-4474-5p with the score of 16 and hsa-miR-559 with the score of 15 (Table 5) (S4 Table). While hsa-miR-146b-5p had a centrality score of 10 it was still described as a breast cancer related miRNA in the literature. hsa-miR-3671 was defined to have both high degree score and expression value in the trastuzumab treated BT474 cells (Table 1). Table 5 showed the detailed information on the most central nodes in both networks.

#### Clustering of the miRNA-miRNA networks clarifies the most regulated biological processes

We performed cluster analyses on the networks to identify tightly connected cliques. MCL cluster algorithm, that considers the weights of the edges, was used to detect the clusters of the network.

In BT474 miRNA-miRNA network, 2.9 were selected as the minimum value for the edge weights to be considered in the clusters. We investigated the first three largest clusters in the network. The clusters comprised of miRNAs with opposite expression values, since the upregulated and downregulated miRNAs regulated the similar pathways.

**Fig 5:**
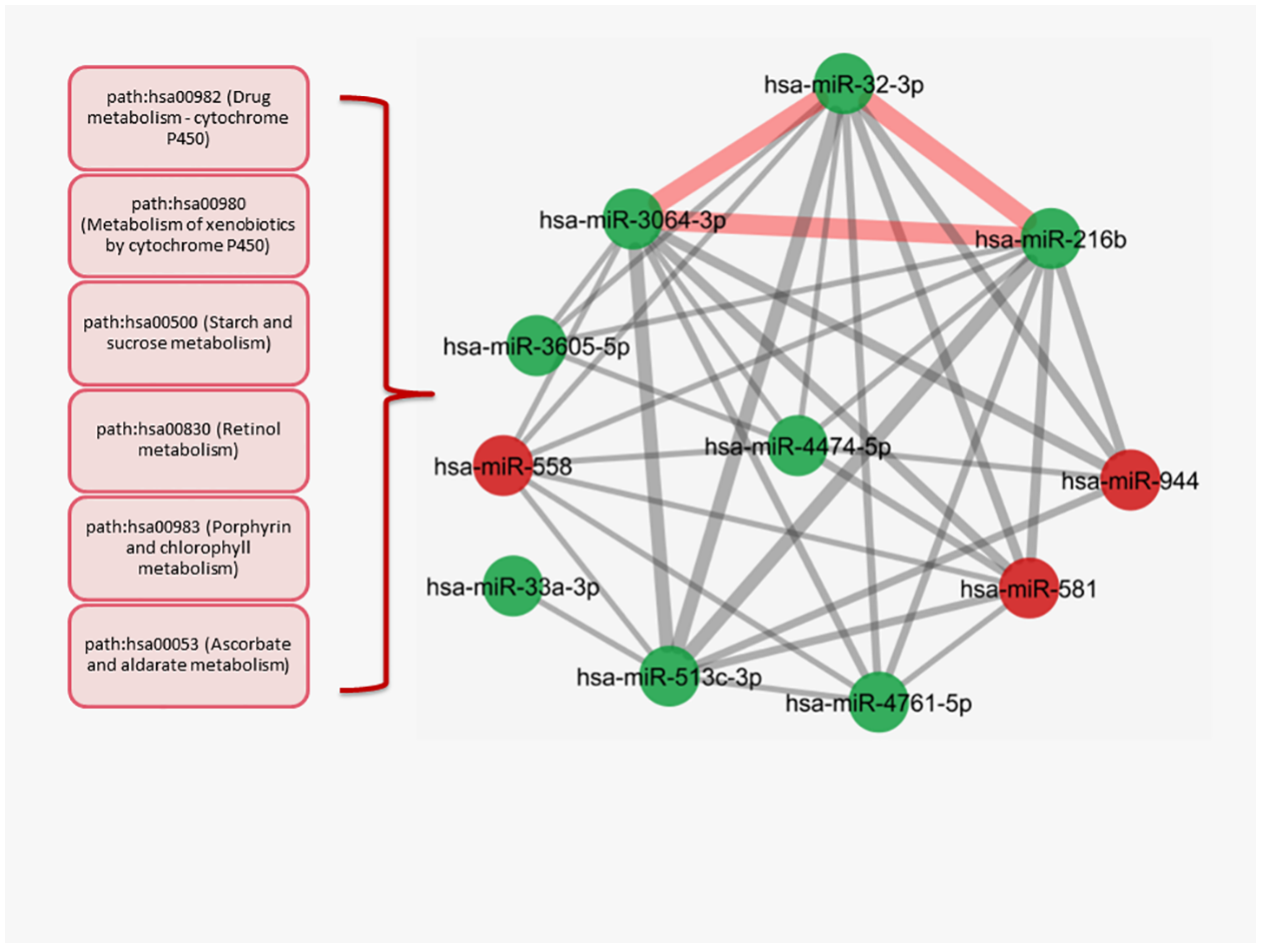
The largest cluster in the BT474 miRNA-miRNA network. The most powerful interaction consisted of the thick edges presented as a triangle (red) between hsa-miR-3064-3p, hsa-miR-32-3p and hsa-miR-216b. The edges are comprised of the metabolic pathways that also dominate the interactions between the other nodes in the complete cluster. The aforementioned pathways were shown in red boxes in left side. (The edge weight minimum value = 2.9, P<0.05 for each miRNA pair, red nodes:upregulated miRNAs, green nodes: downregulated miRNAs).

In the largest cluster (Fig 5), most of the miRNAs gathered around hsa-miR-216b and hsa-miR-3064-3p with strong ties indicating similar biological processes. We also detected a powerful connection between hsa-miR-216b, hsa-miR-3064-3p and hsa-miR-32-3p that was illustrated with thick edges indicating that they have more common pathways than the rest of the miRNAs. The rest of the interactions within cluster were generated with the metabolic pathways such as path:hsa00982 (Drug metabolism—cytochrome P450), path:hsa00980 (Metabolism of xenobiotics by cytochrome P450), path:hsa00500 (Starch and sucrose metabolism), path:hsa00830 (Retinol metabolism), which were the main components of the aforementioned triangle (Fig 5)(S5 Table).

**Fig 6:**
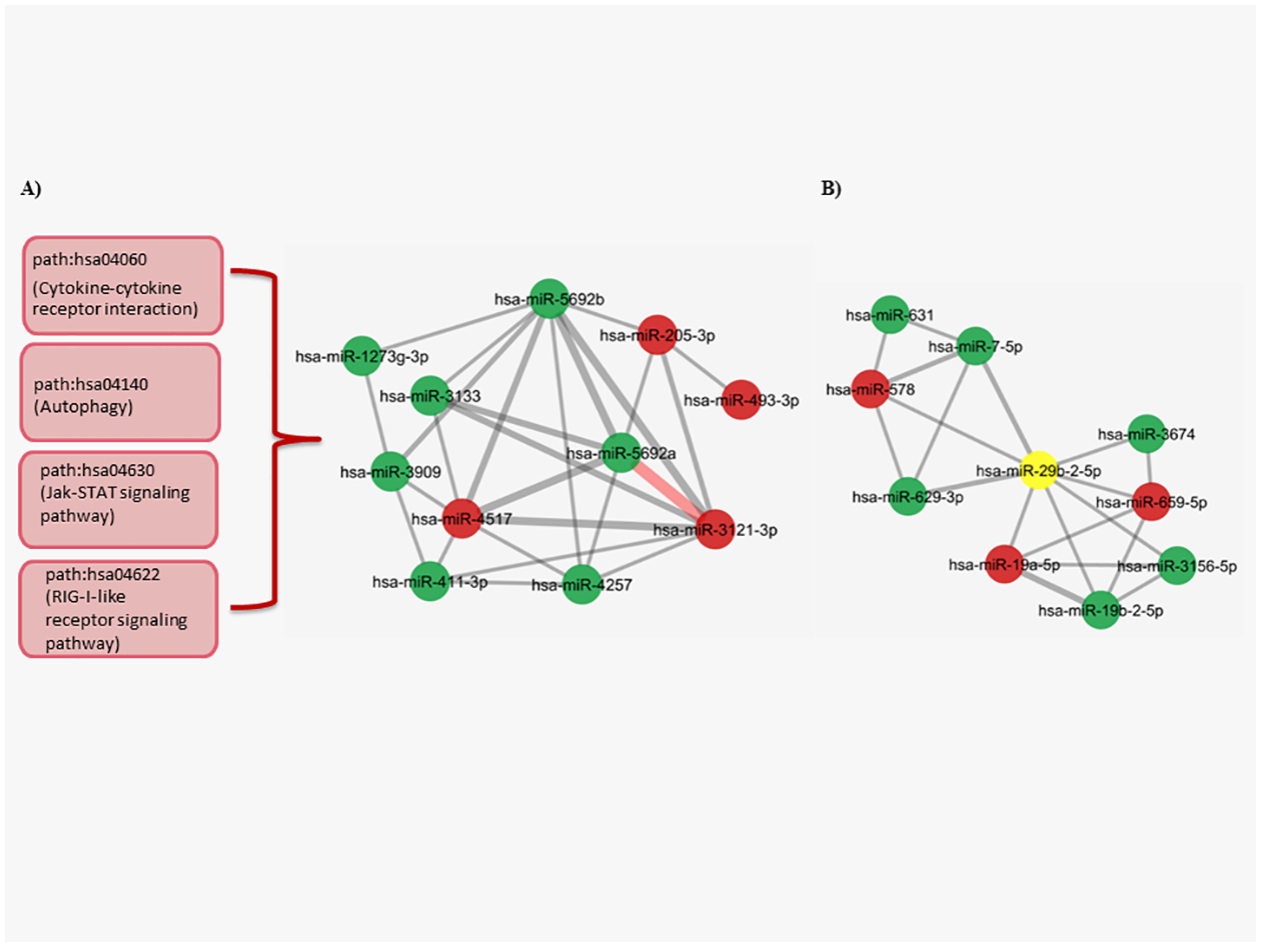
The second and third largest clusters in the BT474 miRNA-miRNA network. (A) The most powerful interaction consisted of one thick edge (red) presented between hsa-miR-5692a and hsa-miR-3121-3p. The edges are made of the cancer related pathways that control majority of the interactions between the other nodes in the complete cluster. The aforementioned pathways are shown in red boxes in left side. (B) In the last cluster, hsa-miR-29b-2-5p (shown in yellow) has important role as a hub node to unite two different groups of miRNAs that enriched in path:hsa05220 (Chronic myeloid leukemia), path:hsa04010 (MAPK signaling pathway) and path:hsa04060 (Cytokine-cytokine receptor interaction), path:hsa04630 (Jak-STAT signaling pathway) pathways (The edge weight minumum value = 2.9, P<0.05 for each miRNA pair, red nodes:upregulated miRNAs, green nodes: downregulated miRNAs).

In the second largest cluster (Fig 6A) the most central node was hsa-miR-4517, which was connected to the other miRNAs through shared pathways. We also observed a strong relationship between hsa-miR-3121-3p and hsa-miR-5692a. This connection and the rest of the total interactions in this cluster were mostly controlled by cancer related pathways such as; path:hsa04060 (Cytokine-cytokine receptor interaction), path:hsa04140 (Autophagy), path:hsa04630 (Jak-STAT signaling pathway), path:hsa04622 (RIG-I-like receptor signaling pathway) (Fig 6) (S5 Table).

In the last cluster (Fig 6B), a known miRNA with roles in various cancers, hsa-miR-26b-2-5p, combined two groups of nodes, which included different shared pathways. The first group was dominated by the pathways such as path:hsa05220 (Chronic myeloid leukemia), path:hsa04010 (MAPK signaling pathway) and it was connected to miRNAs that have functionally enriched in path:hsa04060 (Cytokine-cytokine receptor interaction), path:hsa04630 (Jak-STAT signaling pathway) pathways (S5 Table)(Fig 6).

**Fig 7:**
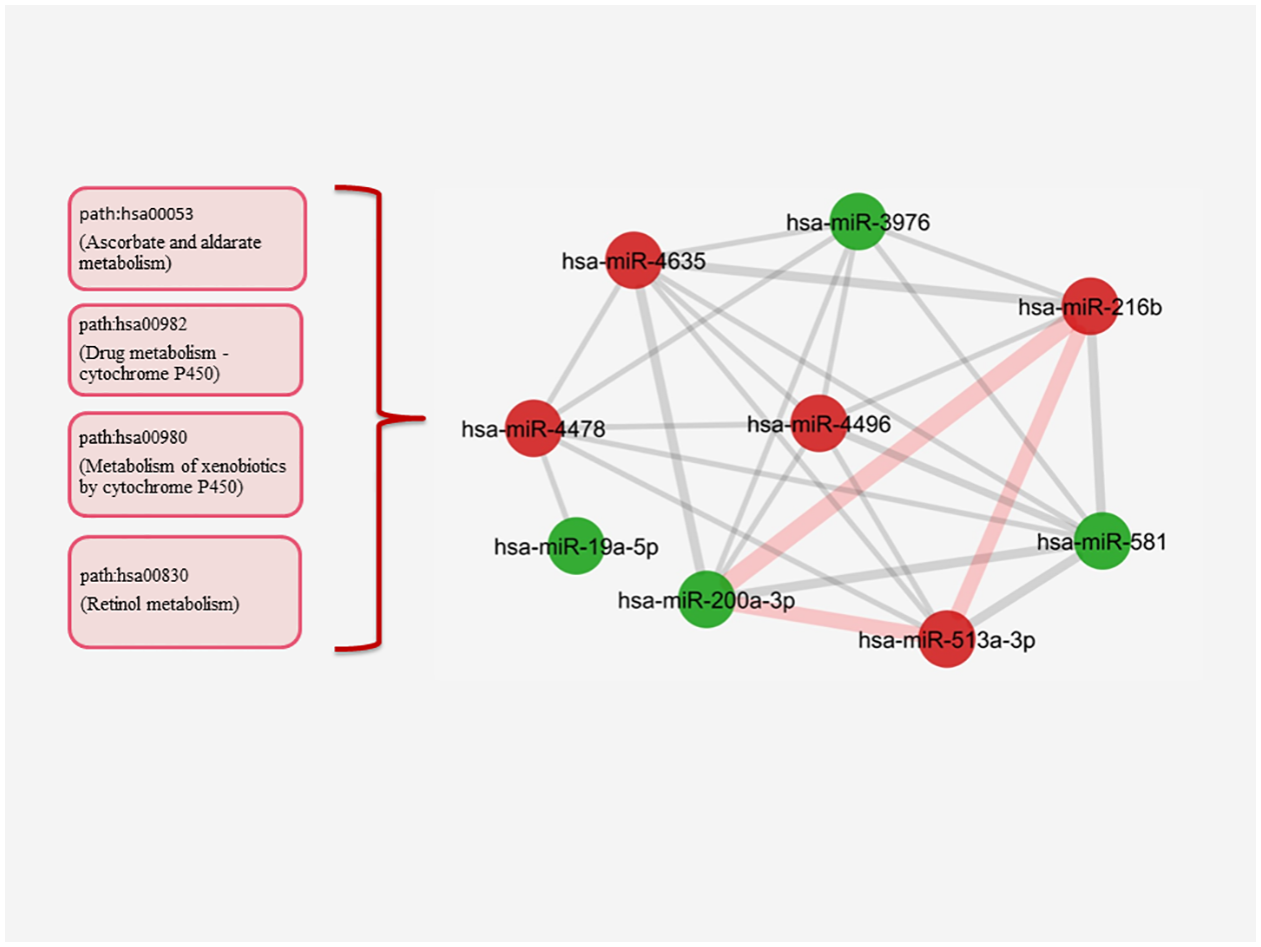
The largest cluster in the SKBR3 miRNA-miRNA network. The most powerful interaction is consisted of the thick edges presented as a triangle (red) between hsa-miR-200a-3p, hsa-miR-513a-3p and hsa-miR-216b. The edges are once again made of the metabolic pathways that also dominate the rest of interactions in the cluster. The afermentioned pathways were shown in red boxes in left side. (The edge weight minumum value = 2.9, P<0.05 for each miRNA pair, red nodes:upregulated miRNAs, green nodes: downregulated miRNAs).

In SKBR3 miRNA-miRNA network, the edge threshold was also set as 2.9. and the three largest clusters were examined in detail. The miRNAs with opposite expression values were also observed together in the clusters of the network. hsa-miR-216b was again the most important node in the first largest cluster (Fig 7). It was connected to its neighbor nodes through the metabolic mechanisms. A strong connection detected between hsa-miR-216b, hsa-miR-200a-3p and hsa-miR-513a-3p was illustrated by thick edges, which consisted of the pathways such as path:hsa00053 (Ascorbate and aldarate metabolism), path:hsa00982 (Drug metabolism—cytochrome P450), path:hsa00980 (Metabolism of xenobiotics by cytochrome P450), path:hsa00830 (Retinol metabolism) (Fig 7) (S6 Table). As it was observed in BT474 cells, this strong connection dominated the other interactions between miRNAs. Distinctively, hsa-miR-216b was defined to be upregulated in SKBR3 cells, while it was downregulated in BT474 cells.

**Fig 8:**
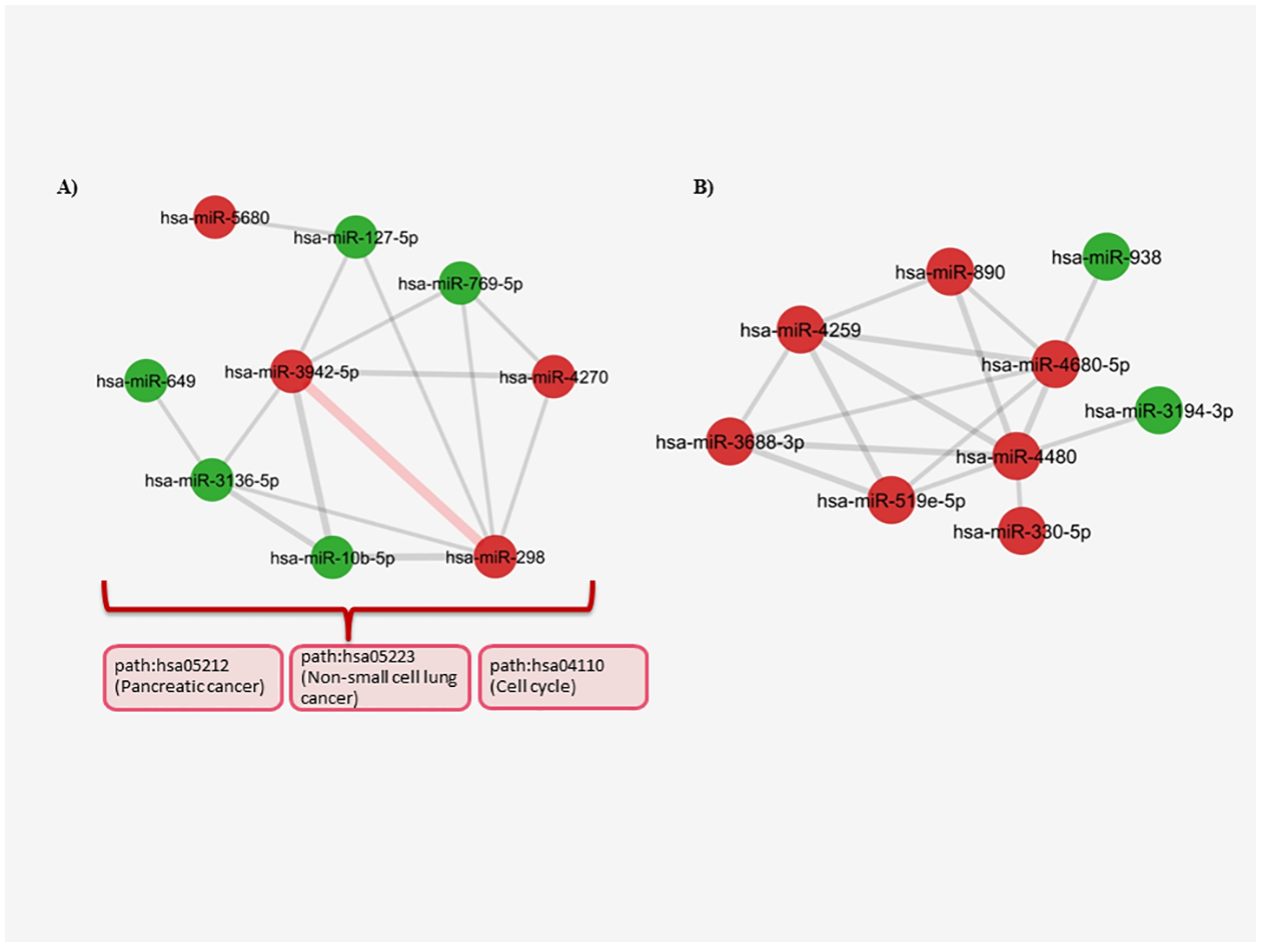
The second and third largest clusters in the SKBR3 miRNA-miRNA network. (A) The most powerful interaction is consisted of one thick edge (red) presented between hsa-miR-3942-5p and hsa-miR-298. The edges are made of the cancer related pathways that control majority of the interactions between the other nodes in the cluster. The afermentioned pathways are shown in red boxes in left side. (B) In the last cluster, the interactions are determined by the upregulated miRNAs mostly and they are related to each other through path:hsa04810 (Regulation of actin cytoskeleton) and path:hsa05200 (pathways in cancer) (The edge weight minumum value = 2.9, P<0.05 for each miRNA pair, red nodes:upregulated miRNAs, green nodes: downregulated miRNAs).

In the second largest cluster (Fig 8A), hsa-miR-3942 was found to be another hub node. It was strongly connected to hsa-miR-298 and hsa-miR-10b through cancer specific pathways such as; path:hsa05212 (Pancreatic cancer), path:hsa05223 (Non-small cell lung cancer), path:hsa05220 (Chronic myeloid leukemia), path:hsa04110 (Cell cycle), and path:hsa04666 (Fc gamma R-mediated phagocytosis) (Fig 8) (S6 Table). We also found out that hsa-miR-10b was one of the miRNAs previously identified to have roles in breast cancer progression. Based upon the pathways shared by hsa-miR-10b, we might lead to a certain point about the functions of its strongly connected neighbors; hsa-miR-298 and hsa-miR-3942 that are presented as novel miRNAs in breast cancer. The rest of the cluster was also strongly controlled by cancer pathways such as path:hsa05212 (Pancreatic cancer), path:hsa05223 (Non-small cell lung cancer), path:hsa04110 (Cell cycle), path:hsa4520 (Adherens junction) underlying the strong effect of two main biological mechanisms that were potentially regulated by responsive miRNAs (Fig 8, S6 Table). In addition, the last cluster (Fig 8B) was consisted of tightly connected upregulated miRNAs and they were related to each other through path:hsa04810 (Regulation of actin cytoskeleton) and path:hsa05200 (pathways in cancer) mostly (Fig 8, S6 Table).

## Discussion

Recent studies showed that miRNA patterns were altered in trastuzumab treatment and associated with drug response. However, our understanding of the miRNA-mediated mechanisms of action in trastuzumab treatment is still very limited, since the previous studies only identified the individual miRNA effects in trastuzumab responsive cell lines rather than explaining the complexity of miRNA-regulatory mechanisms on the systemic level [18–23]. Herein, we focused on discovering the molecular response of the cells to trastuzumab on the level of miRNA-regulatory mechanisms. For this purpose, we constructed a homogenous network model, which enabled us to define the interactions between miRNA pairs by emphasizing the most shared biological processes and pathways that miRNAs were involved in trastuzumab treated cell lines.

In this study, we built a homogenous network model that focuses on the relationships between miRNAs by using pathways that their predicted targets were enriched. The homogenous networks are more applicable to explain the relationships between single types of molecules, which are trastuzumab responsive miRNAs in our case. Unlike heterogenous networks, homogenous networks allow the integration of a property of a biological unit (e.g., pathways of miRNAs) into the network by utilizing them as connectors instead of representing them explicitly as nodes [43]. Our network model not only highlighted the interplay between responsive miRNA pairs but also exposed the functional patterns shared by them. We identified the functional relationships at three different levels; including the investigation of the most targeted genes, the miRNA-miRNA networks built by shared enriched pathways and the clusters of miRNAs with joint functional properties in the network. Integrating different types of analyses increased the significance of synergistic relationships between miRNA pairs.

The input data for the network analysis was obtained by microarray profiling that contained 2006 miRNAs from the updated version of miRBase 19. The profiling was performed in SKBR3 and BT474 cell lines defined as HER2 overexpressing, trastuzumab responsive cell lines. The microarray analysis showed the distinctive expression profiles between trastuzumab and PBS treated breast cancer cell lines. The strong difference in the expression profiles of treated and non-treated cells was consistent with the findings of previous studies [18,19].

We followed a framework that helped us to yield our miRNA-miRNA networks by only focusing on the statistically significant miRNA pairs with the most reliable predicted target genes and enriched pathways. We found out that the most targeted genes in our networks were defined to possess longer 3’UTR binding sites. Additionally, we figured out that majority of them (24 out of top 30 genes in SKBR3 network and 23 out of top 30 in BT474 network) were differentially expressed in invasive breast cancer tissues. In a recent study, Cheng et al. demonstrated the correlation between miRNA regulation and evolutionary conserved genes with longer 3’UTR binding sites [42]. When we consider their potential density of binding sites in the 3’UTR region, those hub genes with high numbers of connection might be important key players of miRNA-mediated regulation in trastuzumab treatment. The literature review also showed that most of them have important roles in cancer progression. Among those, UBE2W plays an important role in the coordination of the attachment of the ubiquitin molecules to the existing proteins. In addition, a literature search showed that FAM9C was identified to have several roles in cancer development such as promoting the tumor growth in the liver, while ARL15 was found to regulate adiponectin levels, which were dysregulated in cancer [44–46]. However, the most targeted gene in both cell lines, SAMD12, was not identified in cancer previously. Nevertheless, it is explicitly take place in the cohort of highly targeted genes; therefore it might potentially have similar functions with the other most targeted genes in the network.

For each network, we investigated the nodes with high degree centralities since they are the key players of the system. In SKBR3 miRNA-miRNA network, hsa-miR-3976 was the most central node and it was followed by miRNAs such as hsa-miR-10b-5p, hsa-miR-190a, which were defined to have important roles in breast cancer metastasis and tumor growth [47,48]. Moreover, in the BT-474 miRNA-miRNA network, hsa-miR-146b-5p was one of the most central nodes and it was described as a potential breast cancer related biomarker in the literature [49]. This showed that our network model was able to capture the prominent miRNAs in both trastuzumab treated cell lines and the presence of the central nodes in the top 20 differentially expressed miRNA list makes them reliable regulatory candidates.

Analyses of the clusters proved that some of the highly related miRNAs were brought together with the help of their common biological processes without providing any additional information. We discovered that the largest clusters were connected through metabolic and cancer related pathways. The most powerful interactions within the first clusters were formed as triangles by certain miRNAs in which most of their edges belonged to metabolic pathways. This might indicate the strong effect of miRNA regulation upon the metabolic machinery in trastuzumab treatment. Among the members of the leading triangles, hsa-miR-216b was an important miRNA in particular, since it was previously associated with breast cancer and found to be common in both cell lines [45]. The interaction shaped by hsa-miR-216b led us to suggest that unknown miRNAs such as hsa-miR-3064-3p and hsa-miR-32-3p of these clusters to be considered as potential candidates with important roles in breast cancer treatment. To clarify the importance of these two miRNAs the trastuzumab responsive genes were identified by in silico analysis in FFPE samples obtained from long-term survivors having early progression to trastuzumab (GSE44272) [50] and three of the responsive genes were found to be common with the potential targets of hsa-miR-3064-3p and hsa-miR-32-3p. These target genes, YWHAE (tyrosine 3-monooxygenase/tryptophan 5-monooxygenase activation protein epsilon), RPL37 (ribosomal protein L37) and AK2 (adenylate kinase 2) were found to have functions in apoptosis, cell cycle and metabolic pathways. These results underlined the regulative roles of two miRNAs on the molecular markers of trastuzumab treatment. Moreover, hsa-miR-26b-2-5p was a hub miRNA linking all the other members of the cluster whose edges were represented by cancer related pathways.

These results might not only clarify the functionally relevant miRNAs in the drug treatment, but also signify the presence of two main biological groups, which are potentially driven by trastuzumab responsive miRNAs; metabolic and cancer related pathways. Further functional characterization of prominent miRNAs from the network analysis by in vitro or in vivo approaches may contribute to the miRNA mediated regulation in trastuzumab treatment. Furthermore different trastuzumab treatment protocols (i.e. incubation time, concentration and sensitivity of the cells) could provide better understanding of the roles of trastuzumab responsive miRNAs in treatment through the comparison of miRNA-miRNA interactions among the various conditions.

## Supporting information

S1 Fig Heatmap of differentially expressed miRNAs in trastuzumab treated BT474 cells. (TIFF)

S2 Fig Heatmap of differentially expressed miRNAs in trastuzumab treated SKBR3 cells. (TIFF)

S1 Table Differentially expressed miRNAs in trastuzumab treated BT474 and SKBR3 cells. (XLSX)

S2 Table Differentially expressed miRNAs omitted from the final list. (XLS)

S3 Table Differentially expressed miRNAs used for the final list. (XLS)

S4 Table The degree centrality scores of miRNA-miRNA networks in SKBR3 and BT474. (XLSX)

S5 Table The largest clusters in BT474 network and KEGG pathway nomenclature. (XLSX)

S6 Table The largest clusters in SKBR3 network and KEGG pathway nomenclature. (XLSX)
