# Supplementary figures and images for: Construction of miRNA-miRNA networks revealing the complexity of miRNA-mediated mechanisms in trastuzumab treated breast cancer cell lines
Source: PLoS One. 2017 Oct 5;12(10):e0185558. doi: 10.1371/journal.pone.0185558 (PMC5628841; doi:10.1371/journal.pone.0185558)

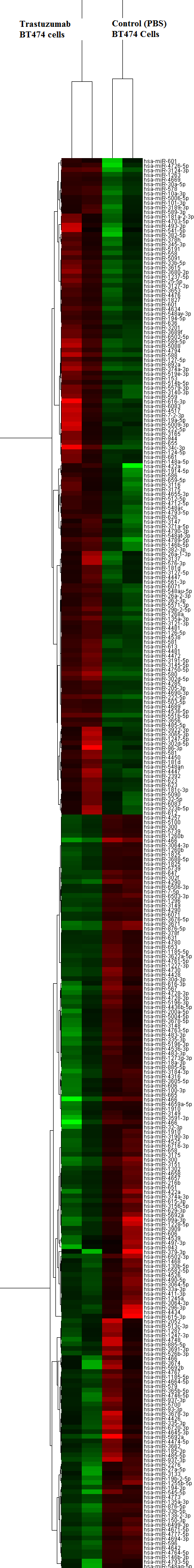

Supplement: S1 Fig — (TIFF) [file pone.0185558.s001.tiff]

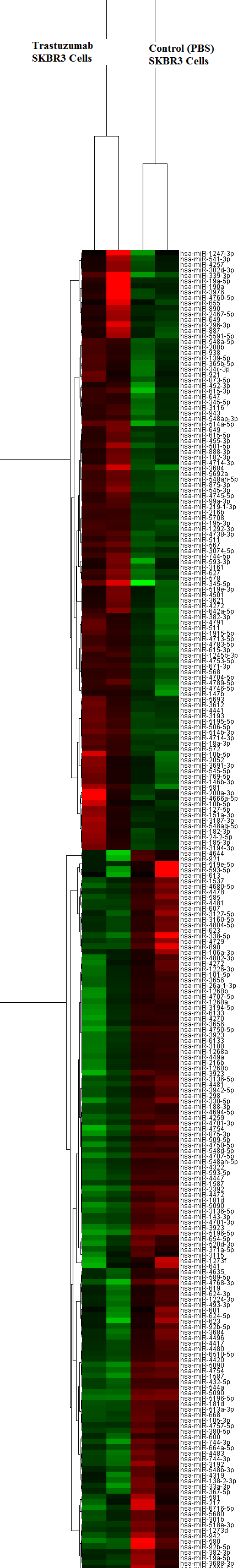

Supplement: S2 Fig — (TIFF) [file pone.0185558.s002.tiff]
